# EVALUATING THE ASSOCIATION BETWEEN SLEEP AND MEMORY IN OLDER VETERANS WITH PTSD

**DOI:** 10.1093/geroni/igz038.1000

**Published:** 2019-11-08

**Authors:** Katherine S Hall, Miriam C Morey, Hayden Bosworth, Jean Beckham, Michelle Pebole

**Affiliations:** Durham Veterans Affairs Health Care System, Durham, North Carolina, United States

## Abstract

Sleep disturbances are core symptoms of posttraumatic stress disorder (PTSD), and recent studies also suggest a link between PTSD and cognitive impairment. There is some evidence of an association between sleep disturbances and cognitive abilities, such as memory, though few studies have focused on older adults and fewer still among those with mental health conditions. This study examined the association between subjective memory complaints and sleep (quality and quantity) in older veterans with PTSD. Fifty-four veterans with PTSD (M age=67.4, 85.2% African American, 90.7% men) participated in the study. Sleep was assessed using the Pittsburgh Sleep Quality Inventory (PSQI) and the PSQI Addendum for PTSD (PSQI-A). Memory was assessed using the Frequency of Forgetting Scale (FOF) derived from the Memory Functioning Questionnaire. The relationship between sleep quality parameters and FOF were examined using bivariate correlations and independent samples t test. Over 60% of participants met military-specific criteria for poor sleep (PSQI≥10; PSQI-A>5). Overall sleep quality on the PSQI-A was significantly associated with worse memory (r=-0.38, p<.01). Among specific sleep parameters (e.g., sleep latency, sleep duration), greater daytime dysfunction due to sleepiness was significantly associated with worse memory (r=-0.44, p<.01). Between-group analyses comparing memory complaints across participants classified as ‘poor’ versus ‘good’ sleepers on the PSQI-A approached significance (t(52)=1.93, p=.06). This study suggests that poor sleep may be a correlate of memory complaints among older adults with PTSD. Future studies are needed to determine whether poor sleep is an underlying factor in the link between PTSD and cognitive impairment.

